# Nano-Domains Produced through a Two-Step Poling Technique in Lithium Niobate on Insulators

**DOI:** 10.3390/ma13163617

**Published:** 2020-08-16

**Authors:** Yuejian Jiao, Zhen Shao, Sanbing Li, Xiaojie Wang, Fang Bo, Jingjun Xu, Guoquan Zhang

**Affiliations:** The MOE Key Laboratory of Weak Light Nonlinear Photonics, School of Physics and TEDA Applied Physics Institute, Nankai University, Tianjin 300457, China; jiaoyuejian@mail.nankai.edu.cn (Y.J.); 2120160179@mail.nankai.edu.cn (Z.S.); 2120180199@mail.nankai.edu.cn (S.L.); xjw@nankai.edu.cn (X.W.); bofang@nankai.edu.cn (F.B.); jjxu@nankai.edu.cn (J.X.)

**Keywords:** lithium niobate, anti-parallel polarization reversal, nano-domain, two-step poling

## Abstract

We proposed a two-step poling technique to fabricate nanoscale domains based on the anti-parallel polarization reversal effect in lithium niobate on insulator (LNOI). The anti-parallel polarization reversal is observed when lithium niobate thin film in LNOI is poled by applying a high voltage pulse through the conductive probe tip of atomic force microscope, which generates a donut-shaped domain structure with its domain polarization at the center being anti-parallel to the poling field. The donut-shaped domain is unstable and decays with a time scale of hours. With the two-step poling technique, the polarization of the donut-shaped domain can be reversed entirely, producing a stable dot domain with a size of tens of nanometers. Dot domains with diameter of the order of ∼30 nm were fabricated through the two-step poling technique. The results may be beneficial to domain-based applications such as ferroelectric domain memory.

## 1. Introduction

Lithium niobate (LiNbO${}_{3}$) is a kind of versatile ferroelectric material, and has been intensively investigated due to its excellent electro-optic [1,2], acousto-optic [3], nonlinear optic [4,5,6,7] properties and possible applications on integrated optics [8] and nonvolatile ferroelectric domain memories [9,10]. Recently, lithium niobate on insulator (denoted as LNOI) [11,12], has attracted much attention because of its potential applications in integrated photonic devices. The domain reversal of lithium niobate films can be achieved by applying a voltage on the conductive probe tip of atomic force microscope (AFM) [13,14,15,16]. As the magnitude and temporal duration of the AFM tip voltage can be controlled precisely to fabricate domain structures with nanoscale position and size precision, domain reversal with an AFM tip voltage is now widely employed in ferroelectric thin films, enabling precise control on the shape and size of the written domains.

Recently, various novel LNOI-based optical elements have been reported, such as electro-optic modulator [17], high-Q microresonators [18], photonic crystals [19,20], ridge waveguides [21] and hybrid lightwave circuits [22]. Besides, another prospective application of LiNbO${}_{3}$ films is nonvolatile ferroelectric domain memories, which stores information based on the polarity of ferroelectric domain. By applying an external electric field, the domain polarization can be switched between two stable states, representing the “1” and “0” bits respectively in binary data storage.

However, several groups have reported an anti-parallel polarization reversal or an anomalous domain inversion effect in various ferroelectric thin films, including BaTiO${}_{3}$ [23], PZT [24,25,26], LiTaO${}_{3}$ [27,28], and LiNbO${}_{3}$ [14,29,30,31,32], and so on. The phenomenon is observed when a ferroelectric film is poled by a large electric field applied to a conductive tip of AFM. The domain polarization right beneath the tip apex is aligned anti-parallel to the external poling electric field, while the domain polarization of surrounding area is aligned parallel to the external poling electric field, therefore, forming a donut-shaped domain pattern. Obviously, such anti-parallel polarization reversal would have a detrimental effect on ferroelectric domain-based applications such as ferroelectric domain memories, which will give wrong information during the readout [33,34,35,36]. In this paper, we proposed a simple two-step poling method to eliminate the anti-parallel polarization reversal, therefore, to generate a stable nanoscale domain element with a uniform polarization distribution within the domain element in LNOI using AFM-tip-based poling technique.

## 2. Materials and Experimental Setup

**Figure 1:**
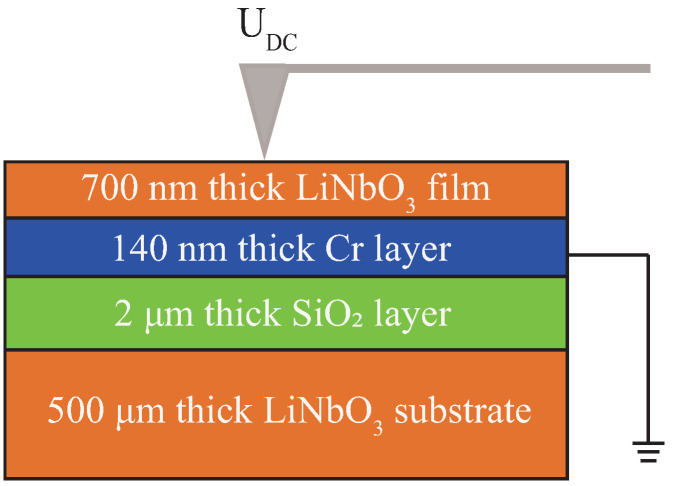
Schematic diagram of the LNOI structure and the domain poling in LNOI with an AFM tip voltage U${}_{DC}$.

Figure 1 shows the schematic diagram of the experimental setup and the layered structure of the LNOI sample used in the experiments. Here, the LNOI sample, from top to bottom, was composed of a +Z-cut ion-sliced LiNbO${}_{3}$ thin film (700 nm thick), a Cr thin film (140 nm thick) as an electrode, a SiO${}_{2}$ layer (2 μm thick), and a LiNbO${}_{3}$ substrate (500 μm thick), which were layered and bonded one to another in sequence. An MFP-3D Infinity atomic force microscope (Asylum Research, Goleta, CA, USA) was employed to fabricate and characterize the domain structures in LNOI. The polarization of the top surface LiNbO${}_{3}$ thin film could be reversed by applying a pulsed square-wave voltage U${}_{DC}$ through the AFM conductive probe tip, as shown in Figure 1, in which the Cr layer was grounded. Then the piezoresponse force microscope (PFM) was employed to visualize the domain distribution with a nanoscale spatial resolution. Here, the tip radius R of the pt-coated Si probe was 20 nm and the resonance frequency *f* of the pt-coated Si probe was 100 kHz, respectively.

## 3. Results

**Figure 2:**
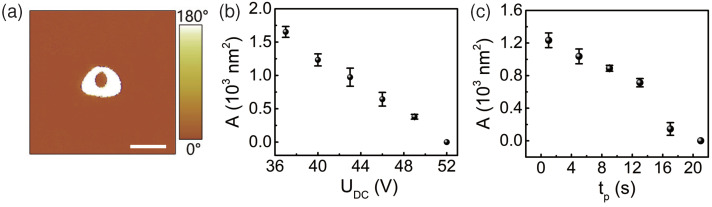
(**a**) is the PFM image of a donut-shaped domain produced with an AFM tip voltage of U${}_{DC}=40$ V and t${}_{p}$ = 1 s. (**b**,**c**) depict the dependence of the inner domain area *A* on the magnitude U${}_{DC}$ and the pulse duration t${}_{p}$ of the tip voltage, respectively. The corresponding pulse duration and magnitude of the tip voltage were set to be t${}_{p}$ = 1 s in (**b**) and U${}_{DC}=40$ V in (**c**), respectively. Scale bar: 100 nm.

For a dot domain written by the AFM tip, its size increases with the magnitude U${}_{DC}$ and pulse duration t${}_{p}$ of the applied tip voltage [13,16,37]. Therefore, a smaller U${}_{DC}$ and a shorter t${}_{p}$ would produce a smaller dot domain, corresponding to a higher data storage density for ferroelectric domain memory. Unfortunately, as several groups reported previously [23,25,31,32], in the case with a short pulse duration t${}_{p}$, although the size of the whole domain will be small, it is possible that the domain polarization just beneath the tip apex is anti-parallel to the poling field, resulting in a donut-shaped domain pattern composed of an outer ring domain and an inner circular domain, as shown in Figure 2a, where the polarization of the outer bright white ring domain is parallel to the poling field, while the polarization of the inner orange circular domain is anti-parallel to the poling field, that is, the polarization of the inner domain was backswitched and parallel to the virgin polarization of lithium niobate thin film. It was observed that the area of the backswitched inner circular domain *A* decreases with the increase of both U${}_{DC}$ and t${}_{p}$, as shown in Figure 2b,c, respectively, and finally the inner domain disappears. These tendencies are in accordance with those reported previously [30]. However, the size of the whole donut-shaped domain will be larger with a larger U${}_{DC}$ and a longer t${}_{p}$, which is not favorable for applications such as domain data storage. In addition, the donut-shaped domain was found to be unstable and it decayed with a time scale of hours, similar to that reported by Shao et al. [38]. Surely, such unstable donut-shaped domain pattern is detrimental for applications such as ferroelectric domain memories.

**Figure 3:**
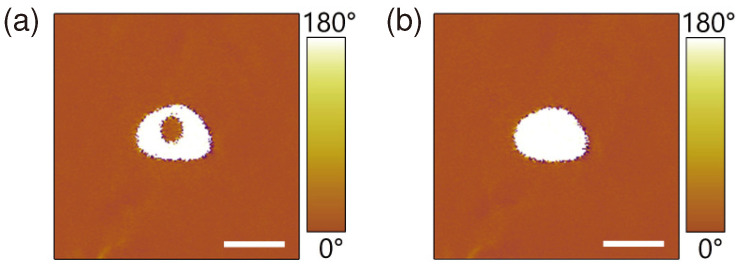
Typical PFM images for domains after the step-1 poling (**a**) and the step-2 poling (**b**), respectively. The magnitude and pulse duration of the poling fields were U${}_{DC1}=40$ V, t${}_{p1}$ = 1 s for the step-1 poling, and U${}_{DC2}=-55$ V, t${}_{p2}$ = 1 ms for the step-2 poling, respectively. Scale bar: 100 nm.

We demonstrated a simple two-step poling technique to eliminate the inner domain while keeping the shape and size of the whole domain unchanged. After the above-described step-1 poling process, a donut-shaped domain was generated, then one applied another poling field on the AFM tip exactly at the same position as that in the step-1 poling stage but with opposite field polarity, this was the step-2 poling stage. With an appropriate magnitude U${}_{DC2}$ and pulse duration t${}_{p2}$ of the step-2 poling field, the central inner domain will disappear while keeping the whole domain shape and size unchanged. Figure 3 shows the typical PFM images of the generated domains after the step-1 poling (a) and the step-2 poling (b), respectively. Here the donut-shaped domain in Figure 3a was produced with a step-1 poling field of U${}_{DC1}=40$ V and t${}_{p1}$ = 1 s, and the magnitude and pulse duration of the step-2 poling field was U${}_{DC2}=-55$ V and t${}_{p2}$ = 1 ms, respectively. One sees from Figure 3b that the inner circular domain in Figure 3a disappears while the shape and size of whole domain is kept unchanged. Moreover, the uniform domain produced after the step-2 poling stage was found to be stable temporally, and no observable decay was detected after a 3-day period.

**Figure 4:**
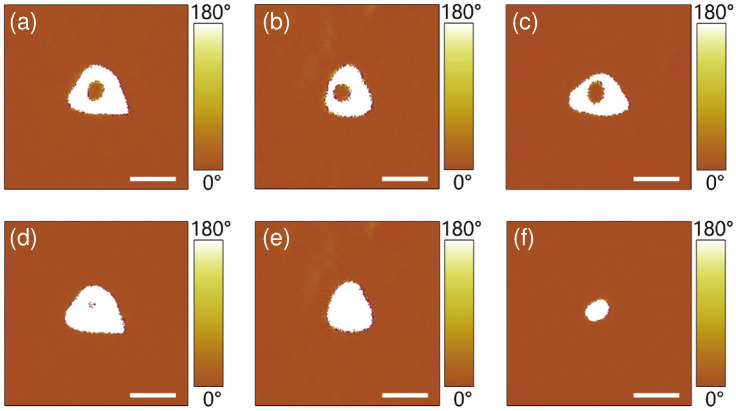
(**a**–**c**) The PFM images of donut-shaped domains fabricated with the same step-1 poling field of $U_{DC1}=40$ V and $t_{p1}=1$ s. (**d**–**f**) The corresponding PFM images of domain structures after the step-2 poling with different field magnitude U${}_{DC2}$ =−51 V, −52 V and −100 V, respectively. The pulse duration of the step-2 poling field was set to be t${}_{p2}$ = 1 ms. Scale bar: 100 nm.

**Figure 5:**
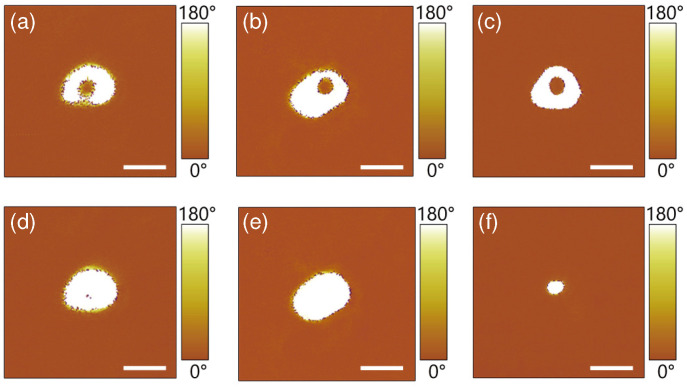
(**a**–**c**) The PFM images of the donut-shaped domains fabricated with the same step-1 poling field of $U_{DC1}=40$ V and $t_{p1}=1$ s. (**d**–**f**) The corresponding PFM images of domain structures after the step-2 poling with different pulse duration t${}_{p2}$ = 0.8 ms, 1 ms and 16 ms, respectively. The magnitude of the step-2 poling field was set to be U${}_{DC2}=-52$ V. Scale bar: 100 nm.

We also studied the effect of magnitude and pulse duration of the step-2 poling field on the reversal efficiency of the inner circular domain. In the experiments, firstly we produced a set of donut-shaped domains with a step-1 poling field of U${}_{DC1}$ = 40 V and t${}_{p1}$ = 1 s, the PFM images of these donut-shaped domains are shown in Figure 4a–c and Figure 5a–c, respectively. Then we loaded the step-2 poling field with different magnitude U${}_{DC2}$ but of a fixed pulse duration t${}_{p2}$ = 1 ms, or with different pulse duration t${}_{p2}$ but of the same magnitude U${}_{DC2}=-52$ V. The typical PFM images of the resulting domain structures are shown in Figure 4 and Figure 5, respectively. Here Figure 4d–f are the cases with U${}_{DC2}=-51$ V, −52 V and −100 V, respectively, while Figure 5d–f show the results with t${}_{p2}$ = 0.8 ms, 1 ms and 16 ms, respectively. One sees that the domain reversal efficiency of inner domain increases with the increase of both U${}_{DC2}$ in Figure 4 and t${}_{p2}$ in Figure 5. The anti-parallel polarization of the inner domain are fully reversed at a critical magnitude U${}_{DC2}=-52$ V in Figure 4 or at a critical pulse duration t${}_{p2}$ = 1 ms in Figure 5. Furthermore, with a larger magnitude or a longer pulse duration of the step-2 poling field, the whole donut-shaped domain will be reversed entirely, resulting in a stable single circular domain with a size of tens of nanometers at the center of the original donut-shaped domain. The diameters of the dot domains in Figure 4f and Figure 5f were measured to be 48 nm and 34 nm, respectively, which is similar to those nano-domains fabricated in LNOI in Refs. [14,16,31,32] and on the surface of the He-irradiated lithium niobate crystals in Ref. [39]. This may provide an effective way to produce nano-scale circular domain, which may be beneficial to high density ferroelectric domain memory. Note that the poling parameters such as U${}_{DC1}$ and U${}_{DC2}$ in both poling steps are dependent on the film thickness. In general, the thicker the film is, the larger the poling voltage should be. In addition, one may also note that, when the nano-domains are writen in series and the distance between neighboring nano-domains is close enough to each other, the subsequently applied poling field may destroy the neighboring nano-domain, therefore, it may become an obstacle to improve the storage density of domain memory. This problem can be partially solved if the nano-domains are written in parallel using this two-step poling technique with an array of AFM tip, that is, one writes a block of nano-domains in parallel with high domain density, but prepares different nano-domain blocks in series while keeping enough space between nearby nano-domain blocks.

## 4. Discussion

**Figure 6:**
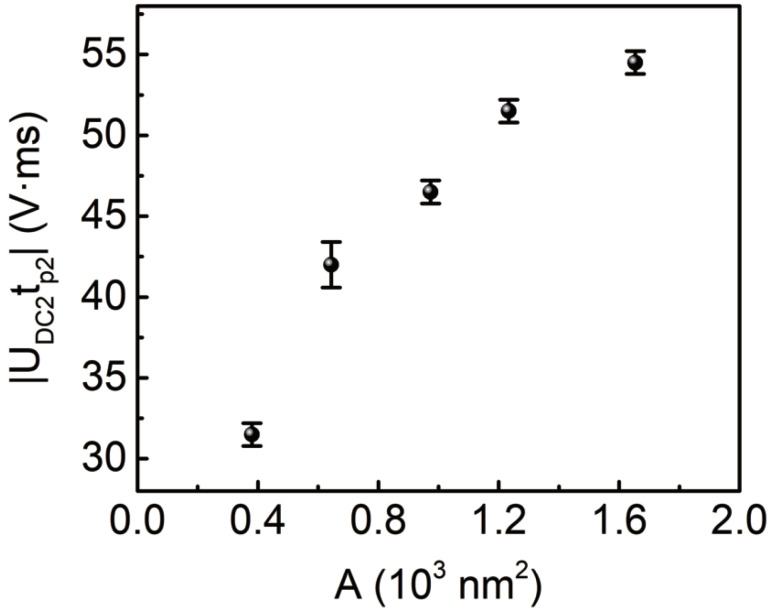
Measured dependence of the product |U${}_{DC2}$t${}_{p2}$| on the inner domain area *A*.

It was reported that the domain poling in LiNbO${}_{3}$ is asymmetric with respect to the crystalline spontaneous polarization, and the coercive field for the forward poling is larger than that of the backward poling, indicating the existence of an internal depolarization field that is antiparallel to the poling field during the forward poling [40,41]. This internal depolarization field is related to the nonstoichiometric defects and relaxes very slowly because it cannot be compensated by the surface charges, and it may result in backswitching effect during the domain poling process under certain conditions [41,42]. Also, charges will be injected into lithium niobate thin film just beneath the AFM tip apex when a strong field is applied through the AFM tip [29,31]. These injected charges will also generate an electric field anti-parallel to the poling field when the AFM tip voltage is removed. On the other hand, the injected charges provide an effective charge compensation for the internal depolarization field, resulting in a reduction of the internal depolarization field as well as the injection-charge-induced electric field. It is the combined action of the internal depolarization field and the injection-charge-induced electric field that result in the observed inner circular domain with its polarization anti-parallel to the poling field. This combined depolarization field decreases with the increase of the injected charge quantity due to the charge compensation process, therefore, the inner domain area decreases with the increase of the magnitude and pulse duration of the step-1 poling field, as shown in Figure 2b,c. In the case when the donut-shaped domain structure is generated after the step-1 poling, and then one loads the step-2 poling field on the inner domain, which is of opposite direction with respect to the step-1 poling field. In this case, the injection-charge-induced field due to the step-2 poling field will reverse its polarity and the inner domain polarization is reversed, as shown in Figure 4e and Figure 5e. It is evident that the injected charge quantity required to reverse the inner domain polarization, which is proportional to the magnitude-pulse-duration product of the step-2 poling field, should increase monotonously with the inner domain area *A*. This was confirmed experimentally, as shown in Figure 6, where the product |U${}_{DC2}$t${}_{p2}$| was the value when the inner domain was just reversed entirely. For convenience, we set the t${}_{p2}$ = 1 ms and varied the field magnitude U${}_{DC2}$ for the data in Figure 6. Similar result was also obtained for the case when one fixed U${}_{DC2}$ while varying t${}_{p2}$. Obviously, the outer ring domain will be backswitched and disappear finally if the step-2 poling field is stronger than the coercive field, and only a bright circular inner domain remains due to the anti-parallel polarization reversal effect.

## 5. Conclusions

In summary, we demonstrated that a donut-shaped domain would be generated due to the anti-parallel polarization reversal effect in LNOI under the action of an AFM tip poling field. We proposed a two-step poling technique to reverse the polarization of the inner domain of the donut-shaped domain. Furthermore, it is possible to fabricate a stable circular domain with a size of tens of nanometers through this two-step poling technique based on the anti-parallel polarization reversal effect, and dot domain with diameter ∼30 nm was produced. This technique may be beneficial to domain-based applications such as ferroelectric domain memory.

## References

[1] Kanno A., Sakamoto T., Chiba A., Kawanishi T., Higuma K., Sudou M., Ichikawa J. (2010). 120-Gb/s NRZ-DQPSK signal generation by a thin-lithium-niobate-substrate modulator. IEICE Electron. Express.

[2] Turner E.H. (1966). High-Frequency Electro-Optic Coefficients of Lithium Niobate. Appl. Phys. Lett..

[3] Courjal N., Benchabane S., Dahdah J., Ulliac G., Gruson Y., Laude V. (2010). Acousto-optically tunable lithium niobate photonic crystal. Appl. Phys. Lett..

[4] Feng D., Ming N.B., Hong J.F., Yang Y.S., Zhu J.S., Yang Z., Wang Y.N. (1980). Enhancement of second-harmonic generation in LiNbO_3_ crystals with periodic laminar ferroelectric domains. Appl. Phys. Lett..

[5] Yamada M., Nada N., Saitoh M., Watanabe K. (1993). First-order quasi-phase matched LiNbO_3_ waveguide periodically poled by applying an external field for efficient blue second-harmonic generation. Appl. Phys. Lett..

[6] Hao Z., Wang J., Ma S., Mao W., Bo F., Gao F., Zhang G., Xu J. (2017). Sum-frequency generation in on-chip lithium niobate microdisk resonators. Photonics Res..

[7] Wang C., Xiong X., Andrade N., Venkataraman V., Ren X.F., Guo G.C., Lončar M. (2017). Second harmonic generation in nano-structured thin-film lithium niobate waveguides. Opt. Express.

[8] Arizmendi L. (2004). Photonic applications of lithium niobate crystals. Phys. Status Solidi (a).

[9] Cho Y., Fujimoto K., Hiranaga Y., Wagatsuma Y., Onoe A., Terabe K., Kitamura K. (2002). Tbit/inch^2^ ferroelectric data storage based on scanning nonlinear dielectric microscopy. Appl. Phys. Lett..

[10] Shur V.Y., Akhmatkhanov A.R., Baturin I.S. (2015). Micro- and nano-domain engineering in lithium niobate. Appl. Phys. Rev..

[11] Levy M., Osgood R.M., Liu R., Cross L.E., Cargill G.S., Kumar A., Bakhru H. (1998). Fabrication of single-crystal lithium niobate films by crystal ion slicing. Appl. Phys. Lett..

[12] Poberaj G., Hu H., Sohler W., Guenter P. (2012). Lithium niobate on insulator (LNOI) for micro-photonic devices. Laser Photon. Rev..

[13] Rodriguez B.J., Nemanich R.J., Kingon A., Gruverman A., Kalinin S.V., Terabe K., Liu X.Y., Kitamura K. (2005). Domain growth kinetics in lithium niobate single crystals studied by piezoresponse force microscopy. Appl. Phys. Lett..

[14] Kan Y., Bo H., Lu X., Cai W., Liu Y., Zhu J. (2008). Growth evolution and decay properties of the abnormally switched domains in LiNbO_3_ crystals. Appl. Phys. Lett..

[15] Ievlev A.V., Jesse S., Morozovska A.N., Strelcov E., Eliseev E.A., Pershin Y.V., Kumar A., Shur V.Y., Kalinin S.V. (2014). Intermittency, quasiperiodicity and chaos in probe-induced ferroelectric domain switching. Nat. Phys..

[16] Gainutdinov R.V., Volk T.R., Zhang H.H. (2015). Domain formation and polarization reversal under atomic force microscopy-tip voltages in ion-sliced LiNbO_3_ films on SiO_2_/LiNbO_3_ substrates. Appl. Phys. Lett..

[17] Wang C., Zhang M., Chen X., Bertrand M., Shams-Ansari A., Chandrasekhar S., Winzer P., Lončar M. (2018). Integrated lithium niobate electro-optic modulators operating at CMOS-compatible voltages. Nature.

[18] Wang J., Bo F., Wan S., Li W., Gao F., Li J., Zhang G., Xu J. (2015). High-Q lithium niobate microdisk resonators on a chip for efficient electro-optic modulation. Opt. Express.

[19] Li Y., Wang C., Loncar M. (2015). Design of nano-groove photonic crystal cavities in lithium niobate. Opt. Lett..

[20] Liang H., Luo R., He Y., Jiang H., Lin Q. (2017). High-quality lithium niobate photonic crystal nanocavities. Optica.

[21] Rabiei P., Steier W.H. (2005). Lithium niobate ridge waveguides and modulators fabricated using smart guide. Appl. Phys. Lett..

[22] Weigel P.O., Savanier M., DeRose C.T., Pomerene A.T., Starbuck A.L., Lentine A.L., Stenger V., Mookherjea S. (2016). Lightwave circuits in lithium niobate through hybrid waveguides with silicon photonics. Sci. Rep..

[23] Abplanalp M., Fousek J., Günter P. (2001). Higher order ferroic switching induced by scanning force microscopy. Phys. Rev. Lett..

[24] Bühlmann S., Colla E., Muralt P. (2005). Polarization reversal due to charge injection in ferroelectric films. Phys. Rev. B.

[25] Kholkin A.L., Bdikin I.K., Shvartsman V.V., Pertsev N.A. (2007). Anomalous polarization inversion in ferroelectrics via scanning force microscopy. Nanotechnology.

[26] Kim Y., Bühlmann S., Hong S., Kim S.H., No K. (2007). Injection charge assisted polarization reversal in ferroelectric thin films. Appl. Phys. Lett..

[27] Moritaa T., Cho Y. (2004). Polarization reversal anti-parallel to the applied electric field observed using a scanning nonlinear dielectric microscopy. Appl. Phys. Lett..

[28] Brugère A., Gidon S., Gautier B. (2011). Abnormal switching of ferroelectric domains created by the tip of an atomic force microscope in a congruent LiTaO_3_ single-crystal thin film. J. Appl. Phys..

[29] Rosenman G., Urenski P., Agronin A., Rosenwaks Y., Molotskii M. (2003). Submicron ferroelectric domain structures tailored by high-voltage scanning probe microscopy. Appl. Phys. Lett..

[30] Kan Y., Lu X., Wu X., Zhu J. (2006). Domain reversal and relaxation in LiNbO_3_ single crystals studied by piezoresponse force microscope. Appl. Phys. Lett..

[31] Lilienblum M., Soergel E. (2011). Anomalous domain inversion in LiNbO_3_ single crystals investigated by scanning probe microscopy. J. Appl. Phys..

[32] Ievlev A.V., Morozovska A.N., Eliseev E.A., Shur V.Y., Kalinin S.V. (2014). Ionic field effect and memristive phenomena in single-point ferroelectric domain switching. Nat. Commun..

[33] Garcia V., Fusil S., Bouzehouane K., Enouz-Vedrenne S., Mathur N.D., Barthelemy A., Bibes M. (2009). Giant tunnel electroresistance for non-destructive readout of ferroelectric states. Nature.

[34] Guo R., You L., Zhou Y., Lim Z.S., Zou X., Chen L., Ramesh R., Wang J. (2013). Non-volatile memory based on the ferroelectric photovoltaic effect. Nat. Commun..

[35] Sharma P., Zhang Q., Sando D., Lei C.H., Liu Y., Li J., Nagarajan V., Seidel J. (2017). Nonvolatile ferroelectric domain wall memory. Sci. Adv..

[36] Jiang J., Bai Z.L., Chen Z.H., He L., Zhang D.W., Zhang Q.H., Shi J.A., Park M.H., Scott J.F., Hwang C.S. (2018). Temporary formation of highly conducting domain walls for non-destructive read-out of ferroelectric domain-wall resistance switching memories. Nat. Mater..

[37] Agronin A., Molotskii M., Rosenwaks Y., Rosenman G., Rodriguez B., Kingon A., Gruverman A. (2006). Dynamics of ferroelectric domain growth in the field of atomic force microscope. J. Appl. Phys..

[38] Shao G.H., Bai Y.H., Cui G.X., Li C., Qiu X.B., Geng D.Q., Wu D., Lu Y.Q. (2016). Ferroelectric domain inversion and its stability in lithium niobate thin film on insulator with different thicknesses. AIP Adv..

[39] Ofan A., Lilienblum M., Gaathon O., Sehrbrock A., Hoffmann A., Bakhru S., Bakhru H., Irsen S., Osgood R.M., Soergel E. (2011). Large-area regular nanodomain patterning in He-irradiated lithium niobate crystals. Nanotechnology.

[40] Peng L.H., Fang Y.C., Lin Y.C. (1999). Polarization switching of lithium niobate with giant internal field. Appl. Phys. Lett..

[41] Gopalan V., Mitchell T.E., Sicakfus K.E. (1998). Switching kinetics of 180 domains in congruent LiNbO_3_ and LiTaO_3_ crystals. Solid State Commun..

[42] Shur V.Y., Rumyantsev E.L., Nikolaeva E.V., Shishkin E.I., Fursov D.V., Batchko R.G., Eyres L.A., Fejer M.M., Byer R.L. (2000). Nanoscale backswitched domain patterning in lithium niobate. Appl. Phys. Lett..

